# Supplementary material for: Changes in health and functioning of care home residents over two decades: what can we learn from population-based studies?
Source: Age Ageing. 2020 Nov 17;50(3):921–7. doi: 10.1093/ageing/afaa227 (PMC8099147; doi:10.1093/ageing/afaa227)
Supplement: aa-20-0910-File002_afaa227 [file aa-20-0910-file002_afaa227.docx]

**Changes in health and functioning of care home residents over two decades: What can we learn from population based studies?**

**SUPPLEMENTARY DATA**

- **Supplementary table 1:** Prevalence, expressed as percentages (with confidence intervals), of severe disability amongst care home residents from 1992-2016 in CFASI/II and ELSA studies. Repeated cross-sectional data for core variables (dressing, bathing, toileting,) and derived variable of severe disability (highlighted in grey).
- **Supplementary table 2:** Prevalence, expressed as percentages (with confidence intervals), of complex multimorbidity (and component variables) amongst care home residents from 1992-2016 in CFASI/II and ELSA studies. Derived variables highlighted in grey. MMSE = Mini Mental State Examination
- **Supplementary Figure 1:** Prevalence of severe disability amongst care home residents from 1992-2016 in CFASI/II and ELSA studies.
- **Supplementary Figure 2:** Prevalence of complex multimorbidity amongst care home residents from 1992-2016 in CFASI/II and ELSA studies.
- **Supplementary Figure 3:** Prevalence of fair/poor self-reported health amongst care home residents from 1992-2016 in CFASI/II and ELSA studies, first observation in care home

Supplementary table 1: Prevalence, expressed as percentages (with confidence intervals), of severe disability amongst care home residents from 1992-2016 in CFASI/II and ELSA studies. Repeated cross-sectional data for core variables (dressing, bathing, toileting,) and derived variable of severe disability (highlighted in grey).

| **Study**  **year** | **CFAS I 1992** | **CFAS I 1994** | **CFAS I 1997** | **CFAS I 2002** | **ELSA**  **2006** | **ELSA**  **2008** | **ELSA**  **2010** | **ELSA**  **2012** | **ELSA**  **2014** | **ELSA**  **2016** | **CFAS II 2010** | **CFAS II 2012** |
| --- | --- | --- | --- | --- | --- | --- | --- | --- | --- | --- | --- | --- |
| **Number in care homes** | 630 | 574 | 256 | 148 | 47 | 62 | 69 | 72 | 59 | 56 | 202 | 105 |
| **Difficulty dressing** | 61.1  (54.8, 67.4) | 66.8  (60.7, 72.9) | 79.8  (72.9, 86.7) | 76.1  (66.6, 85.5) | 73.8  (57.9, 89.7) | 75.7  (61.9, 89.6) | 80.7  (69.4, 92.1) | 87.0  (77.8, 96.2) | 90.0  (81.2, 98.8) | 72.2  (55.9, 88.4) | 84.2  (76.1, 92.3) | 81.7  (71.0, 92.4) |
| **Difficulty bathing** | 72.2  (65.8, 78.6) | 78.5  (72.5, 84.5) | 86.3  (79.6, 92.9) | 87.5  (78.9, 96.0) | 91.3  (81.6, 100) | 88.3  (77.5, 99.1) | 89.5  (81.1, 97.9) | 92.1  (84.2, 100) | 93.3  (87.5, 99.2) | 77.8  (63.6, 92.0) | 76.2  (66.3, 86.0) | 74.1  (61.6, 86.5) |
| **Difficulty toileting** | 54.3  (48.0, 60.7) | 61.0  (54.5, 67.6) | 73.4  (65.9, 80.8) | 65.7  (55.1, 76.2) | 51.9  (33.5, 70.3) | 45.6  (29.7, 61.5) | 62.1  (46.9, 77.3) | 70.7  (57.1,84.3) | 76.7  (64.4, 89.1) | 61.9  (44.4, 79.4) | 88.1  (81.0, 95.2) | 87.3  (78.6, 95.6) |
| **Severe disability** | 63.3  (57.0, 69.6) | 70.1  (64.1, 76.1) | 80.5  (73.5, 87.6) | 75.2  (65.3, 85.1) | 73.1  (57.0, 89.1) | 73.5  (59.2, 87.9) | 81.0  (69.4, 92.5) | 84.5  (74.3, 94.7) | 87.2  (77.2, 97.1) | 69.0  (52.2, 85.8) | 88.9  (82.2, 96.7) | 89.1  (80.6, 97.5) |

Supplementary table 2: Prevalence, expressed as percentages (with confidence intervals), of complex multimorbidity (and component variables) amongst care home residents from 1992-2016 in CFASI/II and ELSA studies. Derived variables highlighted in grey. MMSE = Mini Mental State Examination

| **Study**  **year** | **CFAS I 1992** | **CFAS I 1994** | **CFAS I 1997** | **CFAS I 2002** | **ELSA**  **2006** | **ELSA**  **2008** | **ELSA**  **2010** | **ELSA**  **2012** | **ELSA**  **2014** | **ELSA**  **2016** | **CFAS II 2010** | **CFAS II 2012** |
| --- | --- | --- | --- | --- | --- | --- | --- | --- | --- | --- | --- | --- |
| **Number** | 630 | 574 | 256 | 148 | 47 | 62 | 69 | 72 | 59 | 56 | 202 | 105 |
| **MMSE <20** | 61.5 | 65.5 | 72.0 | 75.2 | - | - | - | - | - |  | 79.0 | 82.4 |
|  | (55.1-67.8) | (59.2-71.8) | (63.2-80.8) | (63.8-86.5) |  |  |  |  |  |  | (68.3-89.7) | (71.9-92.8) |
| **Memory** | 52.9 | 74.9 | 83.6 | 72.4 | 60.5 | 76.1 | 78.6 | 83.5 | 83.1 | 73.0 | 72.9 | 82.3 |
| **problems** | (46.6-59.3) | (69.6-80.2) | (77.5-89.7) | (63.0-81.8) | (43.0-78.0) | (63.4-88.9) | (66.8-90.4) | (73.3-93.7) | (72.9-93.4) | (57.3-88.7) | (62.9-83.0) | (72.0-92.5) |
| **Alzheimer’** | 58.8 | 66 | 80.5 | 74.4 | 18.6 | 35.8 | 36.4 | 41.4 | 44.1 | 33.1 | 77.3 | 77.9 |
| **/Dementia** | (52.9-64.8) | (60.2-71.9) | (73.6-87.5) | (65.0-83.8) | (5.6-31.6) | (20.0-51.5) | (21.0-51.9) | (25.3-57.4) | (25.3-63.0) | (13.8-52.4) | (68.1-86.5) | (66.6-89.2) |
| **Cognitive** | 75.2 | 85.6 | 94.8 | 90.5 | 60.2 | 75.9 | 78.3 | 83.3 | 82.9 | 80.7 | 93.3 | 94.8 |
|  | (69.5-81.0) | (81.3-89.8) | (90.4-99.2) | (84.5-96.5) | (42.5-77.8) | (62.9-88.8) | (66.3-90.3) | (73.0-93.6) | (72.5-93.3) | (67.7-93.7) | (87.9-98.6) | (89.5-100.0) |
| **Cerebrovasc.** | 27.2 | 38.6 | 45.3 | 41.5 | 37.8 | 36.5 | 45.8 | 36.0 | 41.5 | 27.9 | 28.8 | 32.9 |
| **(Stroke)** | (21.5-32.9) | (31.8-45.4) | (35.8-54.8) | (28.0-55.0) | (19.7-55.9) | (20.3-52.8) | (29.9-61.6) | (21.0-51.0) | (23.7-59.2) | (11.3-44.5) | (18.0-39.6) | (19.7-46.2) |
| **Angina** | 9.9 | 20.2 | 31.1 | 21.4 | - | - | - | - | - | - | 11.7 | 12.2 |
|  | (6.4-13.3) | (14.6-25.9) | (22.4-39.8) | (11.9-30.4) |  |  |  |  |  |  | (3.9-19.5) | (3.3-21.2) |
| **Heart** | 10.0 | 19.4 | 23.4 | 16.3 | - | - | - | - | - | - | 10.6 | 13.9 |
| **attack** | (6.5-13.6) | (13.5-25.4) | (15.3-31.4) | (7.7-25.0) |  |  |  |  |  |  | (3.2-18.0) | (4.1-23.7) |
| **Heart** | 16.0 | 28.4 | 41.3 | 26.6 | 36.5 | 35.3 | 45.7 | 57.2 | 56.9 | 53.7 | 23.6 | 33.1 |
| **Problem** | (11.3-20.6) | (21.9-34.9) | (31.7-50.9) | (14.2-39.0) | (19.4-53.6) | (19.9-51.0) | (30.0-61.4) | (42.1-72.4) | (39.4-74.4) | (34.8-72.5) | (13.0-34.2) | (18.4-47.8) |
| **Cardio-** | 21.8 | 36.7 | 49.5 | 36.2 | 36.5 | 35.3 | 45.7 | 57.2 | 56.9 | 53.7 | 30.2 | 40.7 |
| **-vascular** | (16.4-27.2) | (29.5-44.0) | (39.8-59.1) | (39.8-59.1) | (19.4-53.6) | (19.9-51.0) | (30.0-61.4) | (42.1-72.4) | (39.4-74.4) | (34.8-72.5) | (18.4-41.9) | (25.2-56.1) |
| **Musculoskel.** | 50.5 | 63.7 | 72.7 | 71.2 | 38.3 | 53.2 | 49.4 | 64.4 | 42.4 | 39.9 | 48.7 | 67.3 |
| **(arthritis)** | (43.6-57.3) | (57.2-70.3) | (65.3-80.2) | (58.4-84.0) | (21.1-55.5) | (37.2-69.2) | (33.2-65.6) | (49.6-79.3) | (24.6-60.2) | (30.4-69.4) | (37.1-60.4) | (54.4-80.3) |
| **Asthma** | - | - | - | - | 9.1 | 7.7 | 9.4 | 11.5 | 16.2 | 15.1 | 11.8 | 13.9 |
|  |  |  |  |  | (0.0-18.8) | (0.0-15.9) | (1.2-17.5) | (1.5-21.6) | (2.7-29.6) | (0.0-30.4) | (3.7-19.8) | (4.7-23.0) |
| **Bronchitis** | - | - | - | - | - | - | - | - | - | - | 17.5 | 20.4 |
| **(COPD)** |  |  |  |  |  |  |  |  |  |  | (8.6-26.4) | (8.5-32.3) |
| **Lung** | - | - | - | - | - | 5.6 | 9.2 | 10.9 | 12.7 | 11.1 | - | - |
| **disease** |  |  |  |  |  | (0.0-12.0) | (0.0-19.0) | (0.5-21.3) | (0.6-24.8) | (0.0-22.7) |  |  |
| **Respiratory** | 19.8 | 31.2 | 46.8 | 48.0 | 10.8 | 12.4 | 18.8 | 20.2 | 25.2 | 25.6 | 25.5 | 26.3 |
|  | (14.9-24.7) | (24.8-37.6) | (37.5-56.1) | (33.3-62.8) | (0.4-21.2) | (2.4-22.5) | (6.6-30.9) | (7.2-33.1) | (9.0-41.3) | (7.5-43.7) | (15.3-35.6) | (13.9-38.6) |
| **Endocrine** | 12.3 | 16.0 | 23.2 | 11.4 | 12.5 | 16.1 | 27.9 | 27.5 | 37.5 | 19.1 | 15.1 | 15.6 |
| **(diabetes)** | (8.2-16.5) | (10.8-21.1) | (15.0-31.4) | (4.5-18.2) | (1.0-24.1) | (4.1-28.1) | (13.3-42.5) | (12.9-42.1) | (19.3-55.7) | (4.0-34.2) | (6.8-23.3) | (5.9-25.4) |
| **Multi** | 33.3 | 56.5 | 74.8 | 67.4 | 26.2 | 42.0 | 55.1 | 62.9 | 56.0 | 54.0 | 40.8 | 53.6 |
| **Morbidity** | (27.1, 39.5) | (49.5, 63.5) | (67.1, 82.5) | (54.1, 80.6) | (11.1, 41.4) | (25.7, 58.4) | (39.8, 70.8) | (48.2, 77.5) | (37.8, 74.2) | (35.3, 72.8) | (28.5, 53.0) | (39.1, 68.1) |

Supplementary Figure 1: Prevalence of severe disability amongst care home residents from 1992-2016 in CFASI/II and ELSA studies.

Severe disability defined by difficulty in two out of the three domains (washing, dressing, toileting), first observation in a care home.


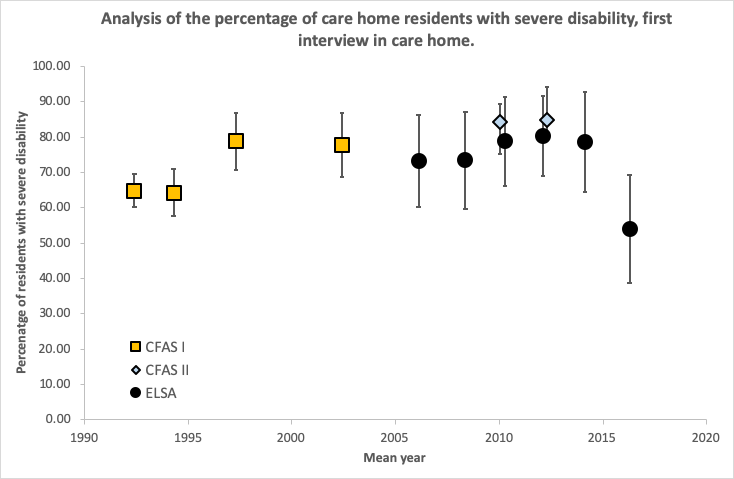


Supplementary Figure 2: Prevalence of complex multimorbidity amongst care home residents from 1992-2016 in CFASI/II and ELSA studies.

Complex multimorbidity defined by medical conditions in at least three out of six domains (cardiovascular, cerebrovascular, musculoskeletal, respiratory, endocrine, cognition), first observation in care home


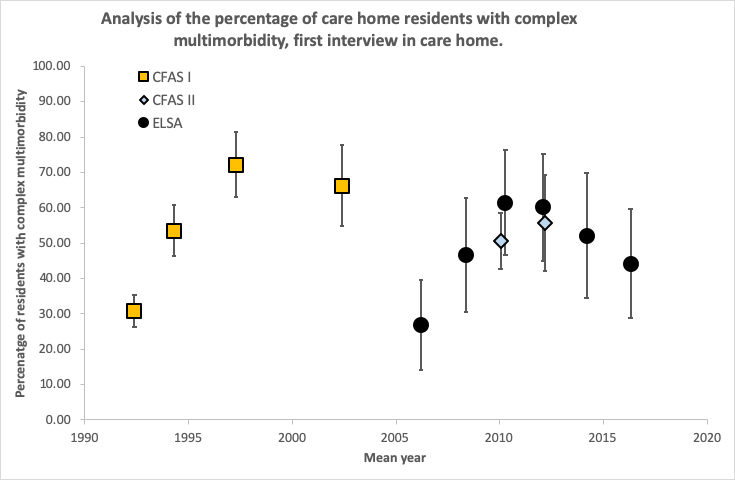


Supplementary Figure 3: Prevalence of fair/poor self-reported health amongst care home residents from 1992-2016 in CFASI/II and ELSA studies, first observation in care home

**
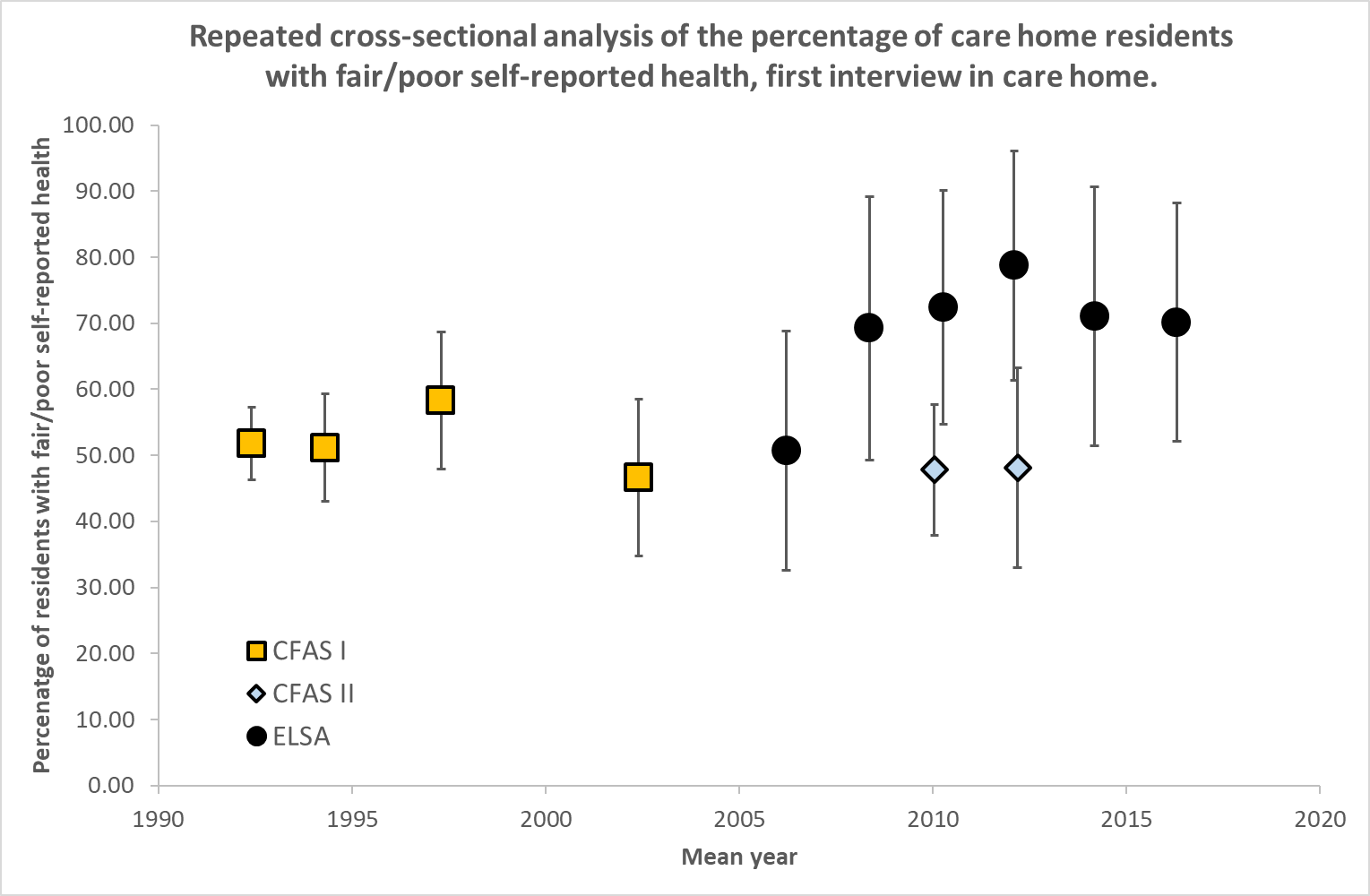
**
